# A short investigation of the effect of the selection of human brain atlases on the performance of ASD's classification models

**DOI:** 10.3389/fnins.2025.1497881

**Published:** 2025-02-05

**Authors:** Naseer Ahmed Khan, Xuequn Shang

**Affiliations:** School of Computer Science and Technology, Changan Campus, Northwestern Polytechnical University, Xi'an, China

**Keywords:** ASD, atlas, fMRI, RS-fMRI, deep learning, pre-processing, classification

## Abstract

This study investigated the impact of brain atlas selection on the classification accuracy of Autism Spectrum Disorder (ASD) models using functional Magnetic Resonance Imaging (fMRI) data. Brain atlases, such as AAL, CC200, Harvard-Oxford, and Yeo 7/17, are used to define regions of interest (ROIs) for fMRI analysis and play a crucial role in enabling researchers to study connectivity patterns and neural dynamics in ASD patients. Through a systematic review, we examined the performance of different atlases in various machine-learning and deep-learning frameworks for ASD classification. The results reveal that atlas selection significantly affects classification accuracy, with denser atlases, such as CC400, providing higher granularity, whereas coarser atlases such as AAL, offer computational efficiency. Furthermore, we discuss the dynamics of combining multiple atlases to enhance feature extraction and explore the implications of atlas selection across diverse datasets. Our findings emphasize the need for standardized approaches to atlas selection and highlight future research directions, including the integration of novel atlases, advanced data augmentation techniques, and end-to-end deep-learning models. This study provides valuable insights into optimizing fMRI-based ASD diagnosis and underscores the importance of interpreting atlas-specific features for an improved understanding of brain connectivity in ASD.

## 1 Introduction

Autism Spectrum Disorder, commonly known as ASD, is a human brain disorder characterized by lack of communication skills, impairment in cognitive abilities, repetitive behavior, and restrictive social interaction (American Psychiatric Association et al., 2013). It is referred to as a “spectrum" because of the wide range of characteristics in the subjects who suffer from ASD. The global burden of ASD is also significant, as ASD has been found in 204 countries worldwide (Solmi et al., 2022) across diverse groups of communities. A comprehensive review (Qiu et al., 2020) of 12 studies consisting of more than 2.0 million people found the prevalence of ASD in Asia to be 0.36%.

### 1.1 Resting state functional magnetic resonance imaging (rs-fMRI)

**Figure 1:**
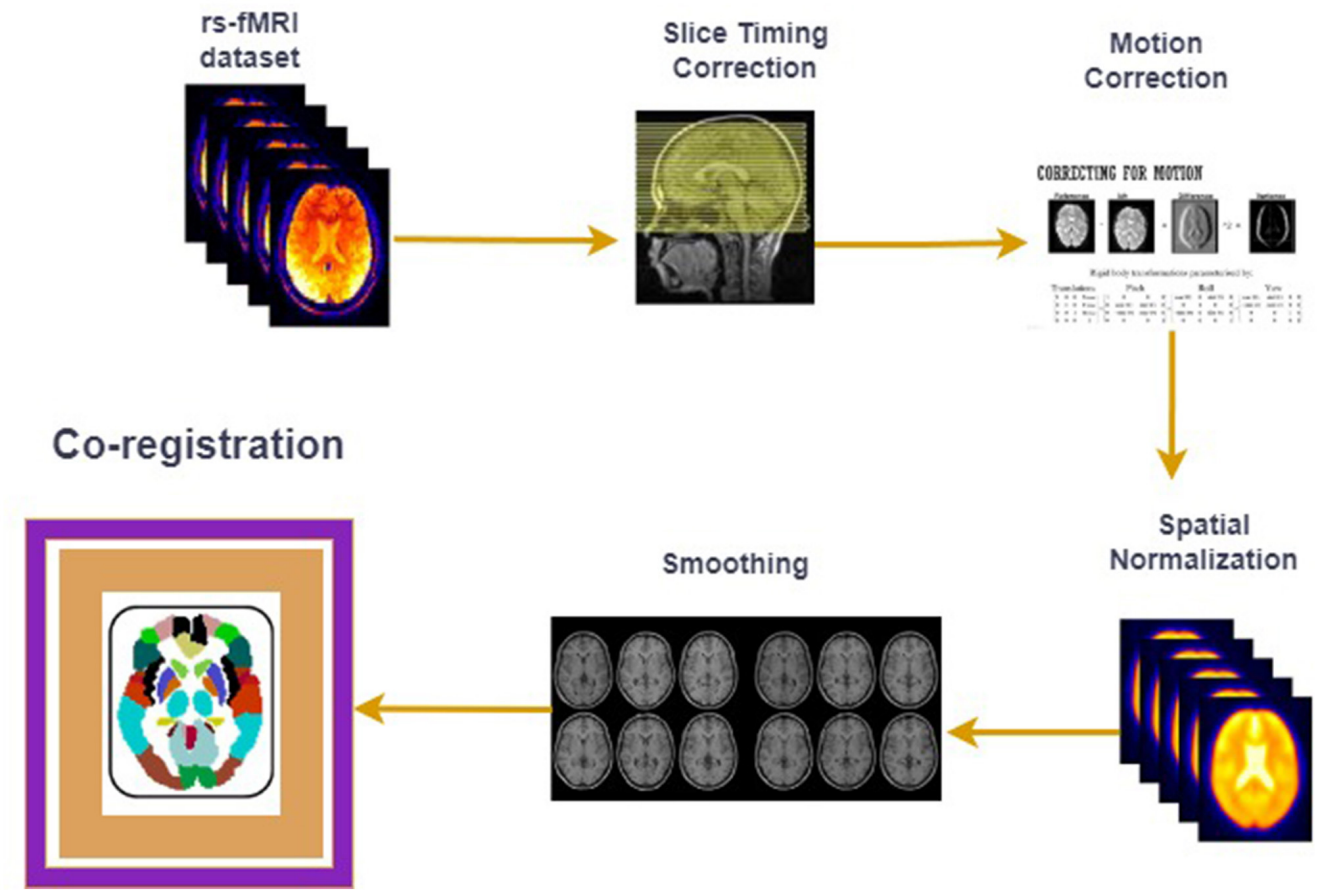
fMRI preprocessing pipeline.

fMRI studies (Walsh et al., 2021; Zhang et al., 2020; Liu et al., 2021) on human brain disorders are getting popular recently as they are being used in a range of human brain disorders such as ASD, ADHD (Tang et al., 2018), schizophrenia (Hoptman et al., 2024) and Alzheimer's (Lajoie et al., 2017). The fundamental idea behind fMRI is to study the Blood Oxygenation Level Dependent (BOLD) signal from the voxels (the volume of the human brain is divided into smaller cubes to extract signals) of the human brain, which acts as a proxy for the original neuronal activity in a particular region. Resting-state fMRI studies (Cole et al., 2010; Santana et al., 2022; Van Den Heuvel and Pol, 2010) are different from task-based fMRI studies because in these studies, subjects did not perform any task, and the BOLD signal measured in this case extracts the intrinsic and spontaneous activities of the human brain. The raw fMRI dataset acquired from the human subject's brain is 4D, as it consists of a 3D set of images taken across the time dimension, therefore, there is a preprocessing stage that follows after acquiring the fMRI data on a particular brain disorder. These preprocessing steps include slice time correction, motion correction, normalization, co-registration, and noise removal. Some of the most crucial preprocessing steps are shown in Figure 1 where the “co-registration" step, in which the human brain is mapped to the known brain atlases and the Regions of Interests (ROIs) are selected, is the main area of focus of the current short investigation.

### 1.2 Brain atlases

Brain atlases are essential tools in neuroimaging studies and serve as reference frameworks that divide the brain into distinct regions of interest (ROIs). These atlases facilitate the study of connectivity patterns and neural dynamics by enabling the standardized analysis of fMRI data. In the context of Autism Spectrum Disorder (ASD), brain atlases provide critical insights into underlying neural disruptions, allowing researchers to systematically examine abnormalities in brain connectivity that are characteristic of the disorder. These brain atlases are used to standardize and simplify the process of analyzing complex brain data using neuroimaging techniques, such as fMRI. Brain atlases can be categorized into two classes, “Anatomical” and “Functional” where the anatomical atlas is related to the physical structures of the brain and the functional atlas is related to the connectivity patterns and functional networks in the brain. The commonly used human brain atlases used in the fMRI prep-processing pipeline are listed below: Each human brain is anatomically different, and without an atlas, it would be difficult to compare brain regions across different individuals. These listed atlases segment the brain into regions of interest (ROIs) based on structural (e.g., AAL Atlas Tzourio-Mazoyer et al., 2002) or functional characteristics (e.g., Yeo's Networks Yeo et al., 2011), allowing researchers to consistently study specific regions across subjects. Associating an ROI with a group of voxels helps aggregate the data from small units (voxels) into meaningful brain regions, making the analysis more interpretable and reducing noise. Voxels, as already defined, represent small volumetric pixels in the brain, but by grouping them into ROIs, researchers can capture the activity or connectivity of larger, anatomically or functionally coherent brain regions, which is especially useful for understanding how different parts of the brain interact (functional connectivity) and for studying disease-related changes in specific brain areas. It also allows for reproducibility across studies, because the same ROIs can be studied using different datasets. Standardizing analyses using these atlases improves the reliability of findings in fields such as neurodevelopmental disorders, cognitive neuroscience, and brain mapping.

Mapping brain atlases in defining ASD relies on their ability to isolate and quantify alterations in brain connectivity. As discussed in Ribeiro da Costa et al. (2022), Chiong et al. (2013), and Harikumar et al. (2021), disruptions in functional networks, such as the Default Mode Network (DMN), Salience Network, and Social Brain Network, are well documented in ASD. Atlases with finer granularity, such as the CC400, provide high-resolution insights into these networks, allowing researchers to capture subtle variations in connectivity. Conversely, coarser atlases, such as AAL, offer computational efficiency by the summarizing brain activity across broader regions. Both approaches provide complementary insights, underscoring the need to select an appropriate atlas based on a specific research question. The dynamics between different atlases also play a crucial role in the understanding of ASD. Each atlas captures distinct aspects of brain organization, and their selection can significantly influence the outcomes of classification studies; coarser atlases such as AAL may miss fine-grained connectivity details but are less prone to overfitting in small datasets, and denser atlases such as CC400 offer detailed connectivity patterns but require larger datasets and more computational resources. Recent studies (Hasana et al., 2024; Heczko et al., 2023) have suggested that combining multiple atlases may yield a more comprehensive understanding of ASD by leveraging the strengths of both anatomical and functional atlases. For instance, researchers could use a coarse atlas such as AAL to identify broad connectivity disruptions and then apply a dense atlas such as CC400 to examine specific regions in detail. This layered approach could provide a deeper understanding of complex neural interactions in ASD.

Furthermore, the process of mapping brain atlases in ASD studies is closely tied to the choice of machine learning and deep learning methods. Feature-based approaches often rely on predefined connectivity metrics extracted from these atlases, whereas end-to-end learning models can directly leverage raw fMRI data, incorporating atlas-based parcellation (“parcellation" is the process of dividing the brain into smaller regions) as input layers. These methodological differences highlight the need to carefully align atlas selection with the study's objectives and the computational framework employed. The five commonly used brain atlases in the reviewed studies are as follows.

1. Automated Anatomical Labeling (AAL) (Tzourio-Mazoyer et al., 2002), which divides the brain into 116 ROIs.
2. Harvard-Oxford (Desikan et al., 2006) which is anatomical and divides the brain regions into 48 ROIs.
3. The Craddock-200 (Craddock et al., 2012) is functional and divides the brain regions into 200 ROIs.
4. CC400 (Craddock et al., 2012) is functional and divides the brain regions into 400 ROIs.
5. Yeo 7/17 (Yeo et al., 2011) is functional and divides the brain regions into 114 ROIs.

### 1.3 Role of atlases

We now briefly explain the state-of-the-art approaches from the literature, the choice of atlas, and the performance of the proposed approach in relation to the selected atlases. An enumeration of various studies, their methodologies, selection of atlas, results, and limitations are also mentioned in Table 1.

**Table 1:** Related studies on the classification of ASD.

**References**	**Method**	**Atlas**	**Dataset size**	**Results (Accuracy)**	**Limitations**
Chen et al. (2022)	An adversarial approach consisting of Graph Neural Networks, in which the authors extracted not only node features but also edge features	Harvard-Oxford	1,007	74.70%	Extracted edge features although promising but still did not achieve promising results
Zhang et al. (2024)	An unsupervised approach that uses shared weights and feature selection	CC200	871	76.52%	Generalization of shared weights is hard and handcrafted feature selection
Khan and Shang (2024)	A GAN inspired approach that uses the architecture of Generative Adversarial based approach to augment the dataset to reduce over-fitting	AAL	884	82.00%	Data Augmentation was based on the connectivity features and not on the original time dimension
Hasana et al. (2024)	Ensemble learning with multi-view fMRI features for ASD diagnosis	Yeo 7/17	871	85.00%	Hand crafted features were used in the first stage
Yang et al. (2023)	GNN with recursive feature elimination for ASD classification	AAL	872	80.00%	Recursive feature selection approach was first used to select features
Qiang et al. (2023)	Behavioral data combined with fMRI for ASD diagnosis	AAL	871	82.10%	Adding behavioral data has a complex interpretation, which is difficult to justify
Ahammed et al. (2021)	DarkASDNet using a CNN for fMRI-based ASD classification	AAL	871	94.70%	A multi-layer CNN model is too simplisitc approach for the challenging task of ASD classification. Did not report results on site wise comparison which are considered more challenging
Shao et al. (2021)	DCNNs for ASD diagnosis using fMRI and structural MRI data	Harvard-Oxford	1,200	79.50%	Feaures ranking base hand crafted features and Did not report results on site wise comparison which are considered more challenging
Almuqhim and Saeed (2021)	DStacked autoencoders for fMRI-based ASD classification	Craddock 200	1,035	76.00%	A first stage Autoencoder based approach followed by a DNN is hard to tune
Sherkatghanad et al. (2020)	CNN model for fMRI-based ASD classification	AAL	800	70.20%	CNN based multi-layer approach is considered too simplisitic as authors did not report site wise results
Liu et al. (2020)	Ensemble learning with autoencoders for ASD classification	Yeo 7/17	1,200	76.80%	Computationally intensive ensemble methods.
Eslami et al. (2019)	A correlation and partial correlation based approach	ROI estimation	871	67.00%	ROI estimation using a customized approach is challenging.
Hasana et al. (2024)	A multi-atlas deep ensemble network that integrates multiple brain atlases of fMRI data through a weighted deep ensemble network.	AAL, CC200 and EZ	1,035	75.20%	Only reported results on the whole dataset
Heczko et al. (2023)	Develops an automated system leveraging a novel lightweight quantized one-dimensional Convolutional Neural Network (Q-CNN) model to analyze fMRI data, incorporating federated learning for data privacy.	AAL, CC200 and DOSENBACH	1,112	83.70%	ROI estimation using a customized approach is challenging

In Chen et al. (2022), the authors used adversarial graph neural networks for fMRI feature extraction and ASD classification. They applied the Harvard-Oxford atlas to 900 subjects from the ABIDE and HCP datasets and achieved 83.1% accuracy. The approach was based on feature selection, with a focus on robust fMRI feature extraction. One limitation is that adversarial methods may introduce instability in training, require hand-crafted features (hand-crafted features are manually chosen by the researchers, like picking the best), and require careful balancing during optimization.

ASD-SWNet (Zhang et al., 2024) proposed a shared-weight feature extraction and CNN-based classification model for ASD diagnosis. Using the CC200 atlas of the ABIDE dataset (871 subjects), an accuracy of 76.52% was achieved. The approach was end-to-end; however, a limitation is the challenge of generalizing shared-weight networks to other neuroimaging datasets.

ASD-GANNET (Khan and Shang, 2024), study used a GAN-inspired model with multi-head attention for ASD classification, based on 884 ABIDE subjects. The AAL atlas was used for parcellation of brain regions. The method was feature based and achieved an accuracy of 82%. One limitation is the complexity of the GAN architecture, which can make training difficult, especially with smaller datasets and augmentation of subjects based on fixed-size connectivity features.

In a previous study (Hasana et al., 2024), the authors applied ensemble learning techniques with multi-view fMRI features to ASD diagnosis using 871 subjects from ABIDE. The Yeo 7/17 Network Atlas was used for segmentation. The approach is feature-based, combining multiple views of the data and achieving 85% accuracy. The main limitation of ensemble methods is that they can be computationally intensive and require large datasets to perform better.

In a previous study (Yang et al., 2023), the authors used a Graph Neural Networks (GNN) with Recursive Feature Elimination (RFE) for ASD classification, using fMRI data from 872 subjects (ABIDE). The AAL atlas was employed, and the approach involved feature selection before classification, achieving 80% accuracy. One limitation is that GNN models tend to be sensitive to the quality of feature selection and require careful tuning to achieve optimal performance.

In a previous study (Qiang et al., 2023), the authors combined behavioral data with fMRI data for ASD diagnosis using 871 subjects (ABIDE). The AAL atlas was used to define these regions. This approach included feature extraction from behavioral data before classification and, improved the overall model accuracy. One limitation is that integrating behavioral data adds complexity to the model, requiring careful feature engineering.

In (Ahammed et al., 2021), the authors developed the DarkASDNet framework, a deep learning approach using CNNs for fMRI-based ASD classification on 871 subjects (ABIDE). The AAL atlas was used for the ROI selection. The method is end-to-end and focuses on the extraction of key features directly from the data. The model achieved 94.7% accuracy, although one limitation was the high computational cost of CNN-based models.

In a previous study (Shao et al., 2021), the authors applied CNNs to ASD diagnosis using both fMRI and structural MRI data. The Harvard-Oxford atlas was used to define the brain regions. The dataset included 1,200 subjects (ABIDE), and the approach was end-to-end. The method achieved high classification accuracy, but a limitation is that multimodal approaches tend to be computationally intensive and require significant data preprocessing.

In Almuqhim and Saeed (2021), the authors developed a stacked autoencoder (SAE) for ASD classification using fMRI data from 1,035 subjects (ABIDE). The Craddock 200 atlas was used for the ROI selection. This approach is feature-based, extracting low-dimensional representations before classification. The method achieved a classification accuracy of 76%. A limitation of autoencoders is that they often require large datasets and careful tuning to avoid overfitting.

In a previous study (Liu et al., 2020), the authors used an ensemble learning method combined with autoencoders for ASD classification from 1,200 ABIDE subjects. The Yeo 7/17 Network Atlas was used for brain region segmentation. This approach involved feature extraction before classification and achieved state-of-the-art accuracy. However, the complexity of ensemble models can lead to overfitting, particularly when the dataset is small.

In a previous study (Eslami et al., 2019), the authors utilized an autoencoder for feature extraction and classification on the ABIDE dataset (871 subjects). The Craddock 400 parcellation atlas was used for the ROI definition. The approach is feature-based, as the autoencoder reduces the dimensionality of the input before classification. It showed a 1% improvement over the baseline accuracy, but a limitation was the computational cost associated with training the autoencoder.

In (Sherkatghanad et al., 2020), the authors used a CNN model for ASD classification from 800 subjects in the ABIDE dataset. The AAL atlas was employed to define the ROIs, and the proposed approach was end-to-end without explicit feature selection. The model achieved 70.2% accuracy. One limitation is that the CNN architecture is extremely simplistic and requires more data for optimal generalization and feature learning.

In Hasana et al. (2024), authors introduced a multi-atlas deep ensemble network that integrates multiple brain atlases of fMRI data through a weighted deep ensemble network. On the ABIDE I dataset, comprising resting-state fMRI data from 17 international research sites, their approach achieved 75.20%. But a major limitation of their approach is lack of results reporting on the site wise comparison.

In Heczko et al. (2023), the authors proposed a novel Multi-Atlas Enhanced Transformer framework (METAFormer) for ASD classification, employing a multi-atlas approach with self-supervised pretraining. On the ABIDE I dataset, including 406 ASD and 476 typical control subjects, they achieved an average accuracy of 83.7% and an AUC score of 0.832. However, a major limitation of their approach was the lack of on-site comparisons.

## 2 Future directions

Based on the short review discussed in the previous sections, the following areas need to be explored by the research community. A brain atlas like AAL can also be referred to as coarser than the brain atlas CC400 because the former has only 116 ROIs as compared to the 400 in the latter case.

1. The performance of the proposed approach should be verified with denser and coarser atlases. If the selection of a coarser atlas such as AAL-116 has better classification performance, then the reasons and the significance of this should be analyzed.
2. The relationship between different atlases should also be explored; for example, the AAL-116 and C200 atlases are different atlases, but if combining these atlases results in better classification performance, the dynamics and significance of this should also be further analyzed. It is highly possible that some regions of the brain require coarser parcellation, such as AAL-116, but other regions need denser parcellation atlases, such as CC400.
3. The effects of parcellation atlases across various sites should also be explored because the ABIDE dataset on ASD is pooled over 17 sites globally. It is highly possible that some sites provide better classification performance when the AAL-116 atlas is selected and some sites might result in better classification performance when the CC400 atlas is selected. If this association is established, then it would be interesting to determine the reasons for these dynamics.
4. The association of atlas selection and dataset size should also be explored not only on the combined dataset but also on the site-wise dataset. This analysis would provide interesting insights into why the selection of the brain atlas is not consistent in these two comparisons.
5. The relationship between the time dimension and the selected atlas should also be explored; for example, the effect of the time dimension and coarser atlas, such as AAL-116, on classification accuracy. If a smaller time dimension with a coarser atlas, such as AAL-116, results in poorer accuracy as compared to the selection of a denser atlas, CC400, with smaller time dimensions, then potential reasons should be investigated from this analysis.
6. Various data augmentation techniques, and a selected brain atlas are related to the classification accuracy of the proposed approach. If data augmentation techniques work better only when a denser atlas such as CC400 is selected, then what can be inferred from this observation?
7. The effect of the selection of the brain atlases on the handcrafted features-based approaches and the end-to-end approaches should also be explored. If the denser atlas such as CC400 results in better classification performance only when the handcrafted features selection approach is used, then what are the potential reasons for this observation?
8. It would also be very interesting to explore how exactly atlases should be combined, should they be stacked, and then the features should be simultaneously selected from different atlases, or should the feature selection process be sequential, that is, first selecting features from AAL-116 and then from the CC400.
9. It should also be interesting to analyze whether there are recent updates in the development of atlases in other brain disorders like Alzheimer's which resulted in better classification performance, which could motivate the research community to come up with some updated atlases ASD.
10. Finally, and most importantly, there should be a genuine effort in developing a web-based tools that researchers can use to map the selected features to the different brain atlases for interpretability. For example, if someone has used an AAL-116-based dataset and got obtained classification results and now wanted to see the interpretation of the brain regions for the selected atlas, the web-based tools should annotate those features on the selected atlas to interpret those features on brain lobes and hemispheres.

## 3 Conclusions

These investigations delineate the importance of the selection of different kinds of human brain atlase effects on the classification accuracy of various proposed algorithms for human ASD. We believe that we have raised some important questions regarding the selection of commonly used brain atlases. Future studies need to analyze not only the classification accuracy of the proposed algorithm but also the intrinsic nature of the parcellation atlases, and there is a need for a dedicated sections in future studies on the ASD classification problem regarding the various dynamics of the chosen brain atlases.
